# A Volunteer Program in Maine to Transport Community Members to Health Care Appointments

**DOI:** 10.5888/pcd17.200085

**Published:** 2020-08-06

**Authors:** Sarah Levin Martin, James Wood, Steven Soule

**Affiliations:** 1 University of Maine at Farmington, Farmington, Maine; 2 Kennebec Valley Community Action Program, Waterville, Maine

## Abstract

Transportation to health care appointments is a well-known barrier for many people, especially people living in rural areas. At the Kennebec Valley Community Action Program (KVCAP), 1 of 8 regional transportation centers in Maine, a robust volunteer program consisting of 93 drivers complements a staff of 45 drivers and 23 office staff members. The volunteers drive approximately 5 to 40 hours per week and have served for an average 4.4 years (range, 1–26 y); their ages range from 23 to 88. The volunteer driver program consists of a volunteer coordinator who communicates with volunteers; staff members who schedule rides; a software application (app) that serves as an interface between the agency and the volunteers as they drive clients to and from medical and social service appointments; regular training; recognition events; and incentives. Most clients have no other transportation option and indicated in informal surveys conducted by KVCAP that they would not attend appointments if the volunteer program were not available. In rural settings, volunteer driving networks provide a viable model to help meet the transportation needs of the population. Recruitment and retention of volunteers is an ongoing effort.

**Summary**

**What is already known on this topic?**

Transportation is a barrier to accessing health care, especially among rural populations. Because of limited resources to overcome this barrier, volunteer drivers are often needed.

**What is added by this report?**

The Kennebec Valley Community Action Program in Maine offers a transportation service that engages 93 volunteer drivers in Somerset and Kennebec counties and provides more than 1,400 rides per day to people with medical and social service appointments throughout the state.

**What are the implications for public health practice?**

Volunteers can comprise a transportation workforce of sufficient quantity, and with training, quality, to help meet the transportation needs of both rural and urban clients.

## The Early Years of the KVCAP Volunteer Driver Program

Transportation is a well-known barrier to health care in this country, especially in rural settings where distances are great and resources are lacking (1–3). Kennebec Valley Community Action Program (KVCAP) in Maine provides citizens of Kennebec and Somerset counties with safe, dependable transportation services. KVCAP has ongoing working relationships with communities and area social service agencies to offer various transportation services to local citizens.

KVCAP employs 68 people in its transportation division, and the volunteer driver program is a key component of the system. KVCAP is one of Maine’s 10 Community Action Agencies. Community Action Agencies were created by President Lyndon B. Johnson in 1964 as part of the Economic Opportunity Act and its War on Poverty (4). The volunteer driver program originated in the mid-1980s as a collaboration between the Maine Department of Health and Human Services’ child protective services and the state’s regional transportation providers to address the problem of case workers spending much of their work time providing transportation instead of tending to their essential case management duties. The program was developed as a cost-effective solution with the understanding that many of the trips would require travel in rural areas and that safety and confidentiality were critical elements of the project. Background checks and training programs were developed to ensure that passengers were placed in vehicles with trained and qualified drivers.

The program was so successful that the model grew to cover general Medicaid and other contract services as a solution to rural and long-distance transportation. Millions of miles are driven each year by the volunteers (Table). The KVCAP model for productive and safe service delivery is used throughout Maine; 7 of 10 Community Action Agencies offer transportation services that rely on the KVCAP model of partnership with state agencies.

**Table:** Use of Kennebec Valley Community Action Program Volunteer Drivers to Transport People Living in Rural Areas to Health Care Appointments, Maine, 2008–2018

Year	No. of Passenger Trips	No. of Service Miles
2008	99,387	5,304,878
2009	97,984	4,975,697
2010	114,710	4,885,782
2011	112,237	5,003,751
2012	106,681	4,906,397
2013	102,461	3,871,648
2014	79,821	2,002,030
2015	102,107	2,770,866
2016	124,391	3,344,399
2017	107,612	3,167,467
2018	93,340	2,826,686
Total	1,140,731	43,059,601
Average	103,703	3,914,509

In 2005, a study showed that people who had family or friends who could provide transportation had more 1.5 times more visits for chronic care than those who did not, and the small number who used public transportation had 4 more chronic care visits per year than those who did not (5). Another study provided evidence that nonemergency transportation was associated with accessing diabetes care appointments; the authors stressed the importance of providing transportation to Medicaid clients with diabetes, especially in rural settings (6). The transportation barrier is also noted for people with cancer who live in rural settings (7). A health impact assessment conducted in New Mexico suggested that providing public transportation would be beneficial but cost-prohibitive in rural settings, citing the primary challenges as 1) long distances to travel, 2) difficulties in scheduling to meet all needs, and 3) poor road and walking conditions for bus stops. The results are applicable to low-income and disconnected rural areas, where access to health, education, and economic opportunities are limited (8). We suggest that a robust volunteer driving program can help overcome these challenges; hence, the objective of our article is to describe the components of such a program.

## The Current Volunteer Driver Program

The current KVCAP volunteer driver program comprises 93 active drivers, each deciding his or her own schedule, which involves from 5 to more than 40 hours per week. The volunteer drivers range in age from 23 to 88. They have from 1 to 26 years of volunteer driving experience, with an average of 4.4 years of volunteer driving for KVCAP. Of the 93 volunteers, most state that they volunteer for the mileage reimbursement ($0.41 per mile). The client rides are scheduled by KVCAP and are for medical and social service appointments. According to an informal survey of clients, most clients do not have any other option for transportation.

### Components of the KVCAP volunteer driving program

**Volunteer coordinator.** A part-time (20 hours per week) coordinator (S.S.) was hired in 2018 to oversee the volunteer driver program. His responsibility is to recruit, train, and supervise the volunteers. For each new volunteer, he submits the paperwork for a background check and a driving-records check and also maintains a log to be sure each driver has an inspected, registered, and insured vehicle. He trains each new volunteer driver on their expected behavior and the use of a software app. He provides all volunteers with a bright yellow sign to post in the front window of their vehicle that identifies them as KVCAP volunteer drivers.

The coordinator is the connection between the KVCAP transportation program and the network of volunteers. He maintains regular email communication with the volunteers, usually with an update or reminder. He reaches out to each as their vehicle is up for registration or insurance renewal.

**Transportation office staff.** KVCAP employs 6 people who work in the intake office taking telephone calls from passengers, health care providers, and drivers (both volunteer and paid). Typically, the volume is 400 to 600 calls per day. These employees enter the trip data into the scheduling software, manage cancellations and change requests, and process new applications for clients requesting transportation assistance. Another team of 10 schedulers and dispatchers develops schedules according to the volunteers’ desired hours and interacts with the paid and volunteer drivers; schedules change frequently. Approximately 1,400 trips are scheduled each day. Additional staff members include billing and reporting personnel, street supervisors who oversee local agency vehicle operations, and customer service representatives who assist with calls about public transportation.

**Software application.** In its earlier years, KVCAP had a paper-and-pencil system for tracking names and addresses of clients and times for pick-up and drop-off. This system required volunteer drivers to drop off their mileage sheets at the KVCAP office. In 2019, the system was replaced by a software app (developed by HB Software Solutions), which eased the transmission of data between the drivers and KVCAP. The schedules are distributed to the volunteer drivers via a KVCAP-provided smartphone app that allows for real-time interaction and information. The app includes a manifest that lists all the assigned clients for a given day, the client’s pick-up address, the time the client is to be picked up, the client’s telephone number, and, if available or appropriate, other notes helpful to the driver, such as the color of the doorway or the name and number of a caretaker that can be called if needed. Google Maps is embedded in the app. The manifest lists each stop in sequence for the day, and it may include picking up a second client and taking both to the same appointment location or to 2 locations in the same town. To protect the privacy of the client, once the client is dropped off and the mileage logged, the contact information is no longer available on the app.

**Volunteer driver requirements.** Volunteer drivers must post the yellow sign in their vehicle and wear a KVCAP photo identification card. The driver uses the app and Google Maps to find each client at a predetermined time and drive the client to their home from a medical or social service appointment or to an appointment from their home. The volunteer must enter the mileage in the app at each stop, and the app captures the time of that entry. After trips are completed, the driver can use the app to confirm that the mileage was captured by selecting a tab on the screen labeled “performance.” At the end of the driver’s schedule, when he or she returns home, one more mileage entry is entered before the driver exits the app and the data are sent to the server.

**Training.** Upon “hiring,” the volunteer driver is trained by the volunteer coordinator. During the year, the driver may have 1 or 2 additional training sessions. In 2019, the volunteer coordinator met with each driver to train them on use of the new app. Volunteers who agreed to drive children attended a group training session on the state requirements for driving children. Not all volunteer drivers drive children — only volunteers who are willing to drive children and have been properly trained.

**Recruitment.** Recruitment is a continuous process; an advertisement (Figure) runs each month in the free newspaper delivered to all residents of Somerset County and beyond (>200,000 homes). Although a newspaper may seem outdated, it is still a viable source of information for older adults (9), who comprise much of the population of rural Maine. The repeated exposure to the same advertisement is effective in building confidence in the program and influencing subsequent behavior, such as becoming a volunteer driver (10).

**Figure:**
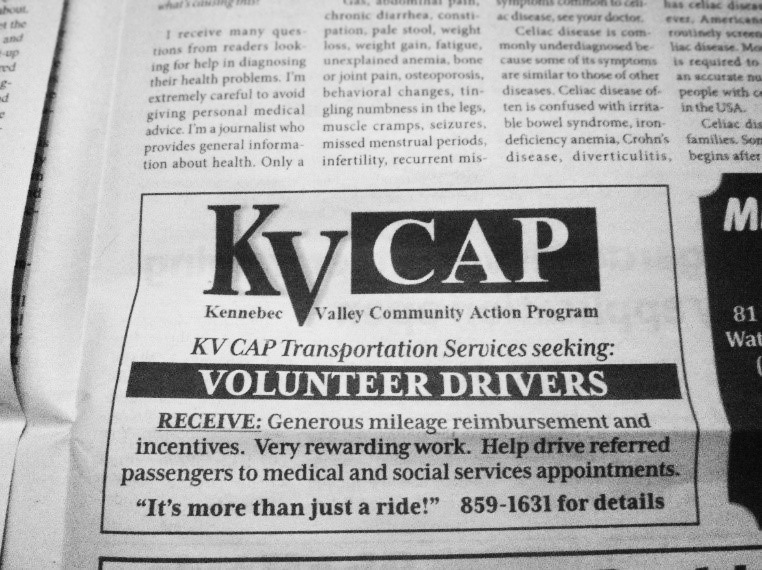
Newspaper advertisement used for recruiting volunteers for the Kennebec Valley Community Action Program’s transportation services in Maine.

The program offers an incentive to volunteers to recruit new volunteer drivers. The monetary incentive was increased in 2020 from $50 to $65 for successful recruitment, which is defined as the new driver volunteers at least 3 days per week and remains a driver for at least 3 months. This incentive was made known to all drivers via email communication from the volunteer coordinator.

**Recognition of volunteers.** Several times each year, the volunteer coordinator emails the volunteers to express appreciation for their work. In 2019, KVCAP hosted a volunteer recognition dinner, which consisted of a welcome from the KVCAP executive director, a historical and statistical summary of the driving program from the transportation director (J.W.), a free dinner, and recognition of each driver who served 1 year, 5 years, 10 years, and so forth. Local businesses donated gift cards (eg, a free oil change, a free pizza) that were raffled at the dinner.

**Reimbursement.** Volunteer drivers are reimbursed $0.41 per mile (covered by Medicare and Medicaid). They receive payments every 2 weeks, which range from approximately $70 to $1,600 per pay period, depending on the driver’s schedule and the distances driven.

### Strengths and weaknesses

The strengths of the volunteer driving program are a low-cost delivery model and efficient resource management. The volunteer model makes great sense for rural transportation needs and long-distance trips when client resources are limited.

The weakness is that volunteers always have the right to refuse trips, and they must assume personal responsibility for vehicle costs, such as fuel, maintenance, and insurance. Short-notice cancellations by riders and drivers create scheduling challenges, and short-notice cancellations by drivers can affect the perception of the program’s reliability among riders. It is also difficult to recruit and retain qualified volunteers.

It is important to note that the volunteer driving program is part of a larger transportation services program at KVCAP, which includes public bus and van transportation. The staffing requirements for the entire division of transportation services at KVCAP are much larger than the staffing requirements for the volunteer driving program and should not be seen as part of model for a volunteer-only network of drivers. Maine has 8 transportation regions, and KVCAP is responsible for the transportation services in 1 of these 8 regions. Given the rural geography of the region, many KVCAP trips cross county lines — beyond the given region — to reach the appropriate providers.

## Lessons Learned, Future Directions

The volunteer driving program at KVCAP is now a robust model; still, recruitment and retention are ongoing challenges as the need for rides increases. During the years of experience rolling out our model, we have found a volunteer program of this size benefits from having a volunteer coordinator. The background checks and training that the volunteer coordinator provides are important factors in the success of the program.

We have also learned that incentives, such as gift cards for positive performance and the volunteers’ help with recruitment are important. Our advertising is ongoing for volunteer drivers, but word-of-mouth among volunteers is a surer route to recruitment. Retention is influenced by volunteer recognition and incentives. Our volunteer recognition event and donated gift cards were much appreciated.

The HB Software Solution app has improved efficiencies. No longer is it necessary for the volunteer driver to drive to the KVCAP office site to drop off their mileage sheet. And mapping is no longer much of a problem because Google Maps is embedded in the app. Some volunteers adapted to the new system with more difficulty than others. The volunteer coordinator played a key role in assisting any driver that needed help with the new app. A year after adoption of the app, the number of scheduling calls from drivers and clients has decreased, and mileage reporting is more accurate.

Volunteer networks will become even more important as Maine seeks to develop resources to meet the increasing demand for transportation, particularly among the growing senior population, who will be looking for transportation resources that enable them to remain at home and maintain their independence. Many rural communities are looking to develop or enhance volunteer driver networks to meet this need. One example is the Neighbors Driving Neighbors program in central Maine (www.neighborsdrivingneighbors.org). Without volunteer drivers, many clients would have no other option for transportation and would not attend appointments. In rural settings, volunteer driving networks provide a viable model to help meet the transportation needs of the population.
